# Generation of physical map contig-specific sequences

**DOI:** 10.3389/fgene.2014.00243

**Published:** 2014-07-22

**Authors:** Yanliang Jiang, Peng Xu, Zhanjiang Liu

**Affiliations:** ^1^Centre for Applied Aquatic Genomics, Chinese Academy of Fishery SciencesBeijing, China; ^2^Aquatic Genomics Unit, The Fish Molecular Genetics and Biotechnology Laboratory, School of Fisheries, Aquaculture and Aquatic Sciences, and Program of Cell and Molecular Biosciences, Auburn UniversityAL, USA

**Keywords:** physical map contig-specific sequences, BAC end sequences, whole genome sequencing, assembly, scaffolding

## Abstract

Rapid advances of the next-generation sequencing technologies have allowed whole genome sequencing of many species. However, with the current sequencing technologies, the whole genome sequence assemblies often fall in short in one of the four quality measurements: accuracy, contiguity, connectivity, and completeness. In particular, small-sized contigs and scaffolds limit the applicability of whole genome sequences for genetic analysis. To enhance the quality of whole genome sequence assemblies, particularly the scaffolding capabilities, additional genomic resources are required. Among these, sequences derived from known physical locations offer great powers for scaffolding. In this mini-review, we will describe the principles, procedures and applications of physical-map-derived sequences, with the focus on physical map contig-specific sequences.

## INTRODUCTION

Whole genome sequencing is the most robust approach to unraveling the genetic information of an organism. During the past several years, sequencing costs have declined drastically through the use of next-generation sequencing technologies, which include a suite of sequencing platforms, such as Illumina sequencing, SOLiD sequencing, and PacBio sequencing, among quite a few others. As a result, more and more species with biological or economic importance are added to the list of organisms whose whole genomes have been sequenced.

However, each of the next-generation sequencing platforms has distinctive shortcomings. For instance, Illumina sequencing and SOLiD sequencing generate accurate but short tags that are difficult to be assembled (Nagarajan et al., 2010; Luo et al., 2012). PacBio sequencing generates long sequences, but its error rate is relatively high. These intrinsic shortcomings affect the assembly qualities of the whole genome sequences, which in turn limit the applicability of these whole genome sequences for genetic analysis.

The assembly qualities of whole genome sequences are measured by a number of parameters including the following: (1) accuracy; (2) completeness; (3) contiguity; and (4) connectivity. Accuracy refers to the correctness of the sequences. It is an important metric as the miscalling of bases can cause substantial trouble for downstream operations, such as the identification of single nucleotide polymorphisms (SNPs). Sequencing accuracy is primarily intrinsic to the sequencing technology but can also be affected by the quality and quantity of the template DNA. In this regard, the Illumina and SOLiD sequencing platforms provide fairly high-quality sequences, while the calling accuracy of the PacBio and Roche 454 sequencing platforms are relatively low. Completeness refers to the percentage of the total bases of the genome that are represented in the assembly of the whole genome sequences. Completeness is important because analysis involving the genes in missing regions will be difficult. Contiguity refers to the lengths of contiguous sequences. Continuous sequences allow full-length gene sequences, including regulatory sequences, to be obtained from the genome sequences for subsequent analysis. Short contigs pose greater challenges for the assembly of the genome into scaffolds, particularly with regards to correct order and orientation. Connectivity refers to the extent to which contigs are properly linked together and reflect their original genomic locations, sequential order, and orientation. For genetic analysis, connectivity is the most important metric. For instance, association analysis has the capability of revealing the significant SNPs associated with a specific trait. If the significant SNPs are located on genome sequences that are well connected at the chromosomal scale, Manhattan plots can be constructed to determine the distribution of significant SNPs along the chromosome(s). The probabilities of the involved significant SNPs can be examined to determine the location of the most significant SNP and how the linkage disequilibrium decays around that specific SNP, thereby determining the number and location of quantitative trait locus (QTLs) involved with the trait. In contrast, if the genome assembly is highly segmented, many significant SNPs remain as isolated contigs or scaffolds, and it will be difficult to determine the number and the location of the QTLs. Therefore, there is limited use for highly segmented genome assemblies (Sierro et al., 2013).

In addition to the intrinsic characteristics of each sequencing technology, the DNA templates used for sequencing can also add additional complexities. Heterozygous diploid organisms with two sets of similar chromosomes pose a challenge for assembly, because it is difficult to distinguish allelic sequences from paralogous loci with high similarities (Hahn et al., 2014). The largest challenge of whole genome sequence assembly most likely comes from the presence of a large number of repetitive elements. Short tandem repeats over 100-bp long often cause a termination of the sequencing reaction, while longer, interspersed repeated sequences prevent short sequence tags from being assembled into long contigs. Repetitive sequences, such as transposons, in the genome shatter *de novo* assembly, because the sequencing reads are usually not long enough to span the entire series of repetitive sequences plus any unique flanking sequences (Jiang et al., 2013). Such challenges are more significant when dealing with species with complex genomes, such as teleost fish, which go through one or two additional rounds of whole genome duplication (Meyer and Van de Peer, 2005; Steinke et al., 2006; Moghadam et al., 2011; Xu et al., 2011b). Assembly is also particularly problematic for species with large genomes. For example, the Norway spruce has a genome size of 20 Gb, and only 25% of its genome is assembled into scaffolds longer than 10 Kb (Nystedt et al., 2013).

Several approaches are available for providing scaffolding capabilities. These include the generation of mate-paired reads from variable lengths of inserts (Boetzer et al., 2011; Gao et al., 2011; Gritsenko et al., 2012; Williams et al., 2012; Hunt et al., 2014; Kajitani et al., 2014; Zimin et al., 2014) or using transcript sequences (Mortazavi et al., 2010). Mate-paired reads can be generated from Illumina sequencing using libraries of various sizes, by using Fosmid libraries (Williams et al., 2012) or bacterial artificial chromosome (BAC) libraries (Xu et al., 2007; Liu et al., 2009). Although extremely efficient, the use of paired reads alone normally cannot reduce the number of scaffolds down to several thousand, as can be done with physical maps. Therefore, we have taken advantage of the available catfish BAC-based physical maps (Xu et al., 2007) and developed a method for generating BAC-based physical map contig-specific sequences (Jiang et al., 2013). Such physical map contig-specific sequences offer the capability to associate all the related genome sequence contigs/scaffolds belonging to a single physical map contig together, effectively reducing the overall number of scaffolds of the genome sequences. Here we will describe the principles, procedures and applications of physical map-derived sequences.

## BAC-BASED PHYSICAL MAPS

A BAC-based physical map consists of contigs of overlapping BAC clone DNA fragments. An acceptable BAC-based physical map usually consists of several thousand contigs. Any gaps can be attributed to missing segments of the genome or to highly competitive regions that cannot be properly assigned to specific contigs. Therefore, physical maps organize the entire genome into several thousand contigs.

Early efforts in whole genome sequencing primarily relied on BAC clones selected from physical maps using a minimal tiling path (MTP, Mahairas et al., 1999; Siegel et al., 1999), and as such, the MTP can be selected through a graph-theoretical approach (Bozdag et al., 2013). Such a sequencing strategy has been referred to as the “clone-by-clone” whole genome sequencing strategy. With this approach, BAC clones selected from the physical map using an MTP are sequenced using random shotgun sequencing and assembly (Lander et al., 2001). The clone-by-clone sequencing strategy reduces the complexity of sequencing and assembly from the genome scale to a BAC clone, thus making it easier to assemble the genome. Such a whole genome sequencing strategy, which utilizes a BAC-based physical map, has been widely used in eukaryotes, such as human (Lander et al., 2001), mouse (Waterston et al., 2002), chicken (International Chicken Genome Sequencing Consortium [ICGSC], 2004), zebrafish (Howe et al., 2013), medaka (Kasahara et al., 2007), *Tetraodon* (Jaillon et al., 2004), *Arabidopsis* (Arabidopsis Genome Initiative [AGI], 2000), and rice (International Rice Genome Sequencing Project [IRGSP], 2005), among many others. However, it is very expensive and labor-intensive, especially for non-model species.

The availability of next-generation sequencing technologies has led to greater efforts in the development of software packages for the assembly of whole genome sequences. However, bioinformatic approaches alone cannot resolve the problems of repetitive sequences, especially with large genomes. As a result, large numbers of contigs have been assembled (reflecting a lower quality) for the whole genome sequences of many species. Further enhancement of the whole genome sequence assemblies is needed to make such assemblies useful. Many scientists have considered coupling traditional approaches with contemporary bioinformatic approaches. As such, physical maps are still crucially useful resources to improve genome assembly, especially for large and complex genomes. For instance, to achieve the assembly of the large barley genome (5.1 Gb), a new strategy was developed to include the construction of a sequence-enriched barley physical map (Mayer et al., 2012). Another important role of physical maps in whole genome sequencing is to orient the assembled contigs/scaffolds. In a pilot study of salmon genome sequencing, a 1-Mb genomic region was sequenced using GS FLX shotgun and long paired-end sequencing, resulting in 175 contigs assembled into four scaffolds, which were then verified and oriented by using a BAC-based physical map and BAC end sequences (BES; Quinn et al., 2008). In another genome sequencing pilot study using catfish, a physical map and BES were used to confirm and order the assembled genome contigs (Jiang et al., 2011). Lewin et al. (2009) concluded that physical maps are indispensable for the precision of genome assemblies, after comparing the quality of the genome assemblies with and without the use of physical maps. Finally, physical maps are essential for assessing the quality of whole genome sequence assemblies (Li et al., 2009; Zhang et al., 2012; Xu et al., 2013; Kim et al., 2014).

## BAC END SEQUENCES

Bacterial artificial chromosome end sequences are genomic survey sequences using BAC clones as templates with sequencing primers from the BAC vector. They are important genome resources, and the most useful BESs are mate-paired reads. As such, BESs have been generated from a large number of species (Budiman et al., 2000; Yuan et al., 2000; Zhao et al., 2001; Larkin et al., 2003; Ren et al., 2003; Messing et al., 2004; Xu et al., 2006, 2011a; Liu et al., 2009).

The use of BESs in whole genome sequencing projects was first proposed as a tool for the identification of MTPs (Goff et al., 2002; Yu et al., 2002). With next-generation sequencing, BESs remain helpful in the assembly and scaffolding process, in particular, for complex and repeat-rich genomes (Feuillet et al., 2011). This is because BESs are paired-reads from large inserts that span a distance of over 100–200 Kb. For instance, the average insert size in the catfish BAC library is 161 Kb (Wang et al., 2007). Mate-paired BESs can be used to combine assembled genome scaffolds into superscaffolds (Quinn et al., 2008; Jiang et al., 2011). Moreover, BESs associated with BAC clones allow them to be related to a physical map, thereby integrating genome sequence contigs/scaffolds with physical maps.

However, a study on catfish demonstrated that BESs are not as powerful as expected when functioning as an anchoring point to link genome contigs to physical maps for two reasons (Jiang et al., 2013): first, BESs are relatively short (Xu et al., 2006; Liu et al., 2009); and second, the number of BESs is still limited because of the high cost associated with generating BESs, and even when all of the BAC clones are sequenced, only two end sequences can be generated per BAC clone. Therefore, additional sequences that are specific for the physical map contigs are needed to enhance the anchoring ability of BAC-associated sequences.

## PHYSICAL MAP CONTIG-SPECIFIC SEQUENCES

Although BESs from physical maps can be used as sequence tags to anchor assembled genome sequence contigs to the BAC contigs of physical maps, they account for only 0.5–1% of all genome sequences. We have developed a simple strategy for the rapid generation of extensive sequence tags from the distinct BAC contigs of physical maps, to allow the vast majority of assembled genome contigs to be anchored to physical map contigs, at a relatively low cost (Jiang et al., 2013).

**FIGURE 1:**
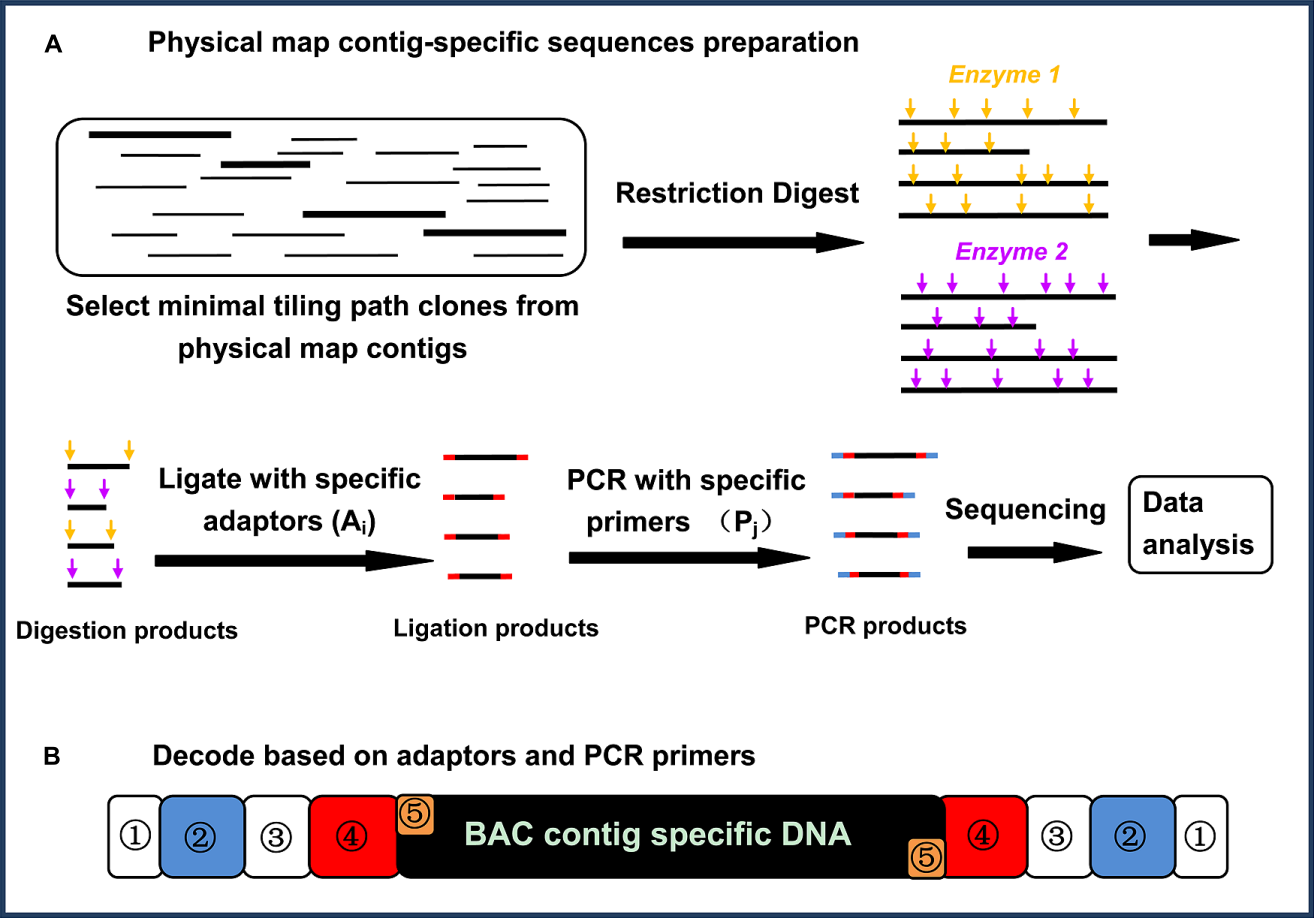
**(A)** A flow chart illustrating the physical map contig-specific fragment preparation. The BAC clones from each physical map contig were selected, using a minimal tilling path, and then the BAC DNA from each physical map contig were pooled for digestion with two restriction endonucleases. In-house designed adaptors were then ligated to the digestion products, followed by amplification using in-house designed primers. All of the PCR products were pooled together and sequenced (Jiang et al., 2013). **(B)** Decoding based on the adaptors and primers. Each PCR product was attached to a physical map contig-specific tag at both ends. The tag representing the physical map contig ID contains five parts: ① represents the shields DNA, ② represents the specific barcoded sequencing in the primer (Pj), ③ represents the common/complementary sequences between the adaptor and the PCR primer, ④ represents the specific barcoded sequences in the adaptor (Ai), and ⑤ represents the overhangs proximal to the fragments for ligation (Jiang et al., 2013)

The core principle of physical map contig-specific sequences is to generate next-generation sequences with known tags specific for each of the BAC contigs in a physical map. Briefly, the strategy for generating physical map contig-specific sequences includes six major steps (**Figure 1A**): (1) select and cultivate the BAC clones from each physical map contig using MTP; (2) extract the BAC DNA, and pool the DNA representing the MTP of each BAC contig from the physical map; (3) digest the DNA by using two 4-bp restriction endonucleases with different recognition sites but compatible overhangs; (4) individually ligate the specific barcoded adaptors to the fragments generated from each BAC contig from the physical map; (5) amplify the specific barcoded fragments via PCR using barcoded PCR primers for the fragments generated from each BAC contig from the physical map; and (6) sequence the PCR-amplified fragments via next-generation sequencing. After sequencing, the sequences can be decoded based on their barcodes to assign them to specific BAC contigs on the physical map.

The highlighted advantages for such a strategy to generate physical map contig-specific sequences are its simplicity and low-cost. A BAC-based physical map normally consists of several thousand BAC contigs, which means that thousands of specific barcodes are required to differentiate each physical map contig-specific sequence. To reduce the total number of barcodes, a two-dimensional tagging strategy was designed, in which there are two separate sets of barcodes; one is attached to the adaptors being ligated to the restriction enzyme digested fragments, and the other is attached to the PCR primer for the amplification of the fragments.

This approach is highly efficient. For instance, we have generated a large number of catfish physical map contig-specific sequences (Jiang et al., 2013) with limited financial resources. The catfish physical map contains 1,824 contigs. If only a single barcode is used, 1,824 specific tags are required. When adopting the two-dimensional tagging strategy, all 1,824 pooled BAC DNA were arrayed into a two-dimensional 38 (row) × 48 (column) setup, using twenty 96-well plates, in which the rows represent one set of tags for adaptors A_i_, where *i* = 1, 2, 3, …38, and the columns represent another set of tags, P_j_, where *j* = 1, 2, 3, …48. As such, each pool of PCR products represents the fragments derived from a single physical map contig with A_i_ and P_j_ at the ends. In this way, only 86 (38 + 48) barcodes are needed, but their combination (38 × 48) can generate 1,824 distinct barcodes. As shown in **Figure 1B**, each end of the amplified fragments attached to the specific barcodes consists of five parts: a common sequence that acts as the “shield” to keep the barcodes intact, the specific barcoded sequence in the primer (P_j_), the common/complementary sequences between the adaptor and PCR primer, the specific barcoded sequences in the adaptor (A_i_), and the overhangs proximal to the fragments to be ligated to the restriction fragments.

**FIGURE 2:**
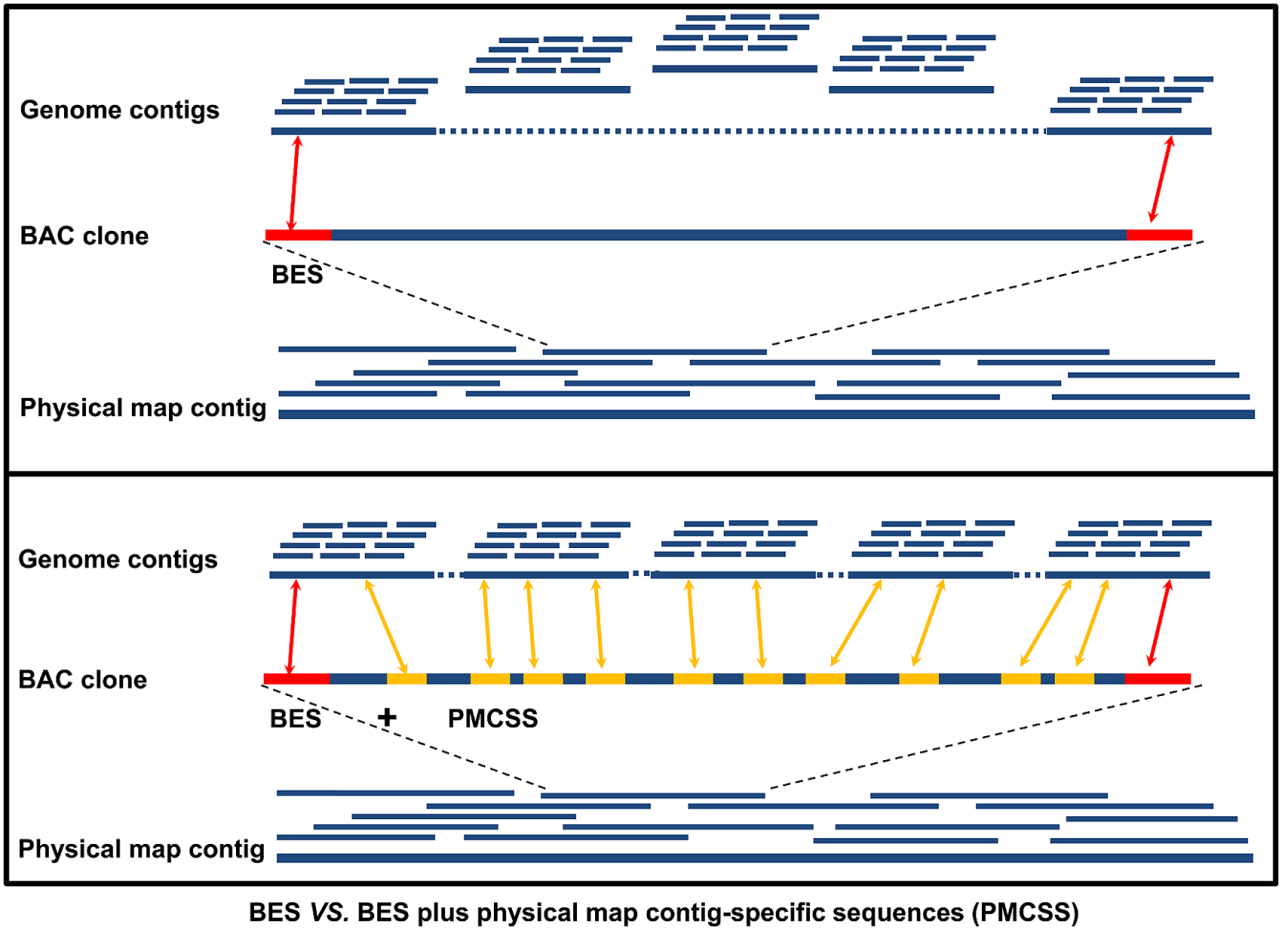
**Comparison of the anchoring power of only BESs versus BESs plus physical map contig-specific sequences (PMCSSs).** The red bar on the BAC clone represents the BESs, while the yellow bar represents the PMCSSs.

One of the most important applications of physical map contig-specific sequences is to associate whole genome sequence contigs into scaffolds. The sequence assemblies obtained from each BAC contig in the physical map can be used to search the contigs in the whole genome sequence using BLAST. Upon receiving hits for two or more of the contigs in the whole genome sequence by one contig in the physical map contig-specific sequences, they are brought together into one contig, thereby reducing the number of contigs in the whole genome sequence. When only one contig from the whole genome sequence is hit, it reveals that the whole genome sequence contig is associated with a specific physical map contig. Therefore, the likelihood of “scaffolding” the contigs in the whole genome sequence is increased. For instance, in our study using catfish physical map contig-specific sequences, we compared the power of anchoring the genome contigs by using BESs alone with using both BESs and physical map contig-specific sequences (Jiang et al., 2013; **Figure 2**). With the previously available BES alone, 27,770 whole genome sequence contigs (11% of the whole genome contigs, channel catfish assembly version 1.0, unpublished) had significant hits to the BESs. When the physical map contig-specific sequences were also used, the number of whole genome contigs with significant hits increased to 156,457. In terms of the total length of the genome contigs being scaffolded, over 79% of the assembled whole genome sequences were anchored when using both BESs and the physical map contig-specific sequences, but only 26% of the assembled whole genome sequences were anchored when only BESs were used. To further assess the scaffolding capacity of the physical map contig-specific sequences, we also determined the number of genes that could be anchored to the scaffolds of the whole genome sequences. The number of genes drastically increased from 6,732 when only BESs were used to 16,680 when both BESs and the physical map contig-specific sequences were used (Jiang et al., 2013). All of these results demonstrated the strong anchoring capability of the physical map contig-specific sequences. However, the order and orientation of the whole genome sequence contigs within the physical map contig is still largely unknown, unless the gaps can be filled by physical map contig-specific sequences.

## CONCLUSION

Next-generation sequencing technologies have provided unprecedented possibilities for genome sequencing. However, challenges remain in generating well-assembled reference genomes due to the short reads produced via the next-generation sequencing platforms and to the complexities of large eukaryotic genomes with high levels of repetitive elements. For genetic analysis, the anchoring of whole genome sequence contigs and scaffolds to chromosomes is perhaps the most important goal. Among the many different approaches for anchoring whole genome sequences to chromosomes, BES and physical map contig-specific sequences provide great power for linking whole genome shotgun sequence contigs to physical maps, thereby significantly reducing the workload when using genetic linkage mapping to anchor whole genome sequence contigs to chromosomes through the integration of genetic linkage and physical maps. The generation of physical map contig-specific sequences is both technologically simple and cost effective.

## Conflict of Interest Statement

The authors declare that the research was conducted in the absence of any commercial or financial relationships that could be construed as a potential conflict of interest.
